# Primary Care Continuity and Wait Times to Receiving Breast Cancer Chemotherapy: A Population-Based Retrospective Cohort Study Using CanIMPACT Data

**DOI:** 10.3390/curroncol28060405

**Published:** 2021-11-17

**Authors:** Rachel Lin Walsh, Aisha Lofters, Rahim Moineddin, Monika Krzyzanowska, Eva Grunfeld

**Affiliations:** 1 Department of Family & Community Medicine, Sunnybrook Health Sciences Centre, Toronto, ON M4N 3M5, Canada; 2 Department of Family & Community Medicine, University of Toronto, Toronto, ON M5G 1V7, Canada; Aisha.Lofters@wchospital.ca (A.L.); rahim.moineddin@utoronto.ca (R.M.); eva.grunfeld@utoronto.ca (E.G.); 3 Department of Family & Community Medicine, Women’s College Hospital, Toronto, ON M5S 1B2, Canada; 4 Dalla Lana School of Public Health, University of Toronto, Toronto, ON M5T 3M7, Canada; 5 Institute for Clinical Evaluative Sciences (ICES), Toronto, ON M4N 3M5, Canada; 6 Princess Margaret Cancer Centre, Department of Medical Oncology & Hematology, University Health Network, Toronto, ON M5G 2C1, Canada; monika.krzyzanowska@uhn.ca; 7 Institute of Health Policy, Management and Evaluation, University of Toronto, Toronto, ON M5T 3M6, Canada; 8 Ontario Institute for Cancer Research, Toronto, ON M5G 0A3, Canada

**Keywords:** breast cancer, primary health care, population health, wait times

## Abstract

(1) Background: Wait times to chemotherapy are associated with morbidity and mortality in breast cancer patients; however, it is unclear how primary care physician (PCP) continuity impacts these wait times, or whether this association is different in immigrants, who experience cancer care inequities. We assessed the association between PCP continuity and the contact-to-chemotherapy interval (wait time from when a patient first presents to healthcare to the first day of receiving breast cancer chemotherapy), with a specific look at the immigrant population. (2) Methods: Population-based, retrospective cohort study of women who were diagnosed with stage I–III breast cancer in Ontario who received surgery and adjuvant chemotherapy. We used quantile regression at the median and 90th percentile to quantify the effect of PCP continuity on the contact-to-chemotherapy interval, performing a separate analysis on the immigrant population. (3) Results: Among 12,781 breast cancer patients, including 1706 immigrants, the median contact-to-chemotherapy interval (126 days) was 3.21 days shorter (95% confidence interval (CI) 0.47–5.96) in symptom-detected patients with low PCP continuity, 10.68 days shorter (95% CI 5.36–16.00) in symptom-detected patients with no baseline PCP visits and 17.43 days longer (95% CI 0.90–34.76) in screen-detected immigrants with low PCP continuity compared to the same groups with high PCP continuity. (4) Conclusions: Higher PCP continuity was not associated with a change in the contact-to-chemotherapy interval for most of our study population, but was associated with a marginally longer interval in our symptom-detected population and a shorter contact-to-chemotherapy interval in screen-detected immigrants. This highlights the importance of PCP continuity among immigrants with positive screening results. Additionally, having no PCP visits at baseline was associated with a shorter contact-to-chemotherapy interval in symptom-detected patients.

## 1. Introduction

Breast cancer is the second most common cause of cancer death in Canadian women [1]. Treating breast cancer often involves surgery and sometimes adjuvant chemotherapy to reduce the risk of recurrence. From 2010 to 2012, 88% of Canadian women with breast cancer received surgery [2], and from 2007 to 2012, 35–41% of Canadian women with stages I–III breast cancer received adjuvant chemotherapy [3]. Increased wait times to receive adjuvant breast cancer chemotherapy were linked with worse morbidity and mortality outcomes [4,5,6,7,8,9,10,11]. Internationally, longer wait times were associated with minority race, older age, comorbidity, rural residence, lower education, stage I breast cancer, mastectomy, gene expression profile testing and being covered through public insurance [12,13,14,15,16,17,18,19,20]. Meanwhile, within Canada and Ontario, shorter wait times were associated with assessment through dedicated breast assessment centres and treatment in South Central Ontario [21,22,23].

Immigrants make up a large proportion of the Ontario population (29.1% of the population according to the 2016 Census) [24], and the association between primary care use and wait times for cancer treatment may be different in this group. Canadian immigrants, despite similar primary care access, are less likely to have their breast cancer detected through screening and experience longer times to diagnosis than long-term residents [25]. In Ontario, immigrants are more likely to be diagnosed with advanced-stage breast cancers than Canadian-born women and are younger at diagnosis [26].

Breast cancer patients frequently visit their primary care physicians (PCPs) during the course of their cancer journey [27]. While PCP involvement in cancer care was shown to increase cancer screening rates, reduce late-stage diagnosis, decrease the use of avoidable hospital and emergency department (ED) visits and improve survival outcomes [28,29,30,31,32], the role of PCPs in affecting wait times to receiving breast cancer treatment is unclear. In qualitative studies and surveys, patients have reported that shorter wait times to cancer diagnosis and treatment are related to PCP responsiveness to patient symptoms, comprehensiveness or breadth of primary care services offered and accessibility of primary care [33,34]. However, a lack of continuity, or a “fresh pair of eyes”, may sometimes shorten the time to diagnosis [35]. The quantitative association between PCP continuity and wait times to receive breast cancer chemotherapy has not been established.

Our study objective was to determine whether PCP continuity was associated with time to chemotherapy. We hypothesised that high baseline PCP continuity is associated with a shorter contact-to-chemotherapy interval (i.e., a shorter wait time from when a patient first presents to healthcare to the first day of receiving adjuvant breast cancer chemotherapy) and that this association is different in the immigrant population.

## 2. Methods

### 2.1. Study Design

We conducted a population-based, retrospective cohort study using linked provincial-level administrative health databases that are housed at ICES, which is a not-for-profit research institute that stores an array of Ontario’s health-related data [36]. This study used data from the Ontario cohort of a larger, nationwide study (the Canadian Team to Improve Community-Based Cancer Care along the Continuum—CanIMPACT) [37].

### 2.2. Study Population

We included women aged 18+ years diagnosed with stage I–III breast cancer from 1 January 2007 to 31 December 2011 (allowing for 5 years of follow-up data for other CanIMPACT studies [38,39]) who received surgery and adjuvant chemotherapy. We excluded patients who had a prior cancer diagnosis or a new primary cancer that was diagnosed within 14 months of the breast cancer diagnosis, had received neoadjuvant chemotherapy or radiation therapy prior to adjuvant chemotherapy or were living in a long-term care facility at the time of diagnosis. Immigrants were defined based on inclusion in the Immigration Refugee and Citizenship Canada permanent resident (IRCC-PR) database, which includes data on immigrants who landed in Ontario from 1985 onward. The remainder of the study population, including Canadian-born citizens or immigrants who landed before 1985, were considered “long-term residents”. Individuals who were identified in the IRCC-PR database were linked deterministically and probabilistically to the registered population of Ontario with an 86% linkage rate [40].

### 2.3. Variables and Data Sources

Our main outcome variable was the contact-to-chemotherapy interval: the number of days from the index contact date (first presentation to any healthcare with a positive mammogram screening or symptoms warranting breast cancer investigation) to the start date of adjuvant chemotherapy. We also looked at two sub-intervals of the contact-to-chemotherapy interval: the primary care interval (from the index contact date to the date of first breast cancer specialist consultation, as defined by the Aarhus Statement used by the International Cancer Benchmarking Partnership (ICBP) [41,42]), and the surgery-to-chemotherapy interval (from the date of last breast surgery to the start of adjuvant chemotherapy).

Our main predictor variable was PCP continuity. Continuity of care is a patient’s experience of coherent and linked care over time. Relational continuity, one aspect of continuity of care, refers to the ongoing relationship between patients and providers [43]. Relational PCP continuity was determined in our study using the Usual Provider of Care (UPC) index, which is a validated measure that is commonly used to assess continuity of care [44,45]: the proportion of visits to the most-often-visited PCP (identified from billings data of ambulatory visits, excluding emergency room visits, to physicians with a ‘General Practitioner/Family Physician’ or ‘Family Physician/Emergency Medicine’ designation) during a 2-year baseline interval (6–30 months prior to the breast cancer diagnosis). We did not calculate the UPC index in patients with fewer than 3 visits to any PCP during the baseline interval since UPC values are less meaningful in this group, where UPC values are limited to 0.0, 0.5 and 1.0. As such, PCP continuity categories in our study were: 0 PCP visits, 1–2 PCP visits, low continuity (UPC ≤ 0.75) and high continuity (UPC > 0.75), as categorised in other studies [25,39,46,47].

We pre-specified potential confounders in our study based on the literature [12,13,14,15,16,17,18,19,20,23] and clinical insight. Potential confounders included the age at diagnosis, neighbourhood income quintile, rurality, physical comorbidity (determined using the Johns Hopkins ACG^®^ System Aggregated Diagnosis Groups (ADGs [48]) and excluding psychosocial ADGs), mental health history (determined by having a mental health visit to a PCP during the baseline period [49]), health region (of which Ontario has fourteen) and primary care practice type (determined by patient enrolment in a particular funding model at diagnosis: ‘team-based capitation’, ‘enhanced fee-for-service (FFS)’, ‘capitation’, ‘straight FFS’ and ‘other’). Patients were considered to be screen-detected if their earliest test within 6 months prior to diagnosis was a documented mammogram screening, or a bilateral mammogram with additional mammogram and/or breast ultrasound ordered by a radiologist the same day or performed on a different day with no other tests that day. Otherwise, the patient was classified as symptom-detected. Databases used to obtain data elements are shown in Appendix A, Table A1. These datasets were linked using unique encoded identifiers and analysed at ICES.

### 2.4. Statistical Analysis

We used chi-squared tests to compare the nominal demographic characteristics across PCP continuity groups. We used Wilcoxon rank-sum tests and Kruskall–Wallis ANOVA to compare the median interval lengths across demographic characteristics and reported the 90th percentile intervals. We performed multivariable quantile regressions at the median and 90th percentile intervals by examining the association between baseline PCP continuity and interval length while adjusting for potential confounders. The contact-to-chemotherapy and primary care intervals were stratified via cancer detection method. We repeated our quantile regression analyses on the immigrant-only population. The few (*n* < 6) patients with implausible interval lengths or missing index contact dates were excluded from our multivariable analyses. All analyses were performed using SAS software, version 9.4 [50]. All *p*-values < 0.05 were considered statistically significant.

### 2.5. Ethics Approval

We obtained approval from the University of Toronto research ethics board.

## 3. Results

There were 12,781 women in our cohort (Table 1), including 1706 Canadian immigrants. Those with no baseline PCP visits (*n* = 800, 6.3%) were more likely to live in remote rural locations, be in the lowest two income quintiles and be diagnosed with stage II/III (versus stage I) disease. Those with low PCP continuity (*n* = 3914, 30.6%) were more likely to be <40 years old, live in urban areas, be immigrants, have more comorbidities and have symptom-detected breast cancer. High PCP continuity (*n* = 6531, 51.1%) was associated with age >60 years, being enrolled in a primary care model and screen-detected cancers.

**Figure 1:**
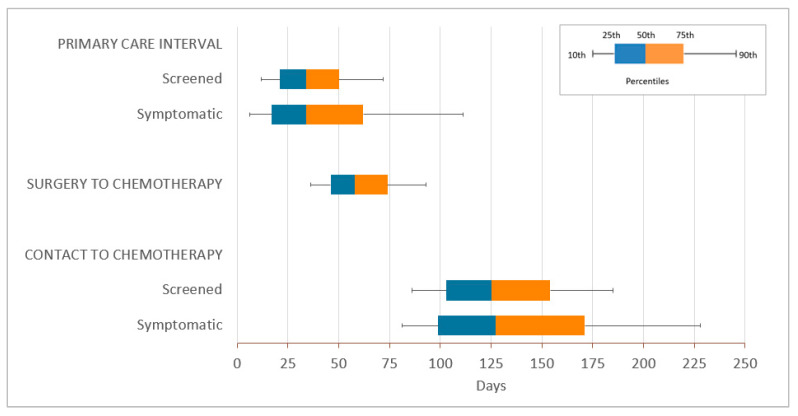
Boxplots of all intervals in days separated by the method of breast cancer detection. Note: Surgery-to-chemotherapy interval not separated by detection method since breast cancer detection is not relevant during this interval.

The median contact-to-chemotherapy interval was 126 days (Figure 1; Table 2). This median interval was 7–12 days longer in those >74 years old and 12–18 days shorter in those <40 years old. The contact-to-chemotherapy interval varied by health region. Women in the Champlain health region had 19–21-day-longer median intervals. Those in the Waterloo Wellington health region had 6–15-day-shorter intervals. Within the screen-detected group, longer intervals were seen in rural areas, with 30-day-longer median intervals in very remote rural neighbourhoods compared to urban neighbourhoods. Within the symptom-detected group, longer intervals were seen in immigrants by 7 days, high comorbidity groups by 12 days and those with a mental health history by 7 days. Among immigrants, the median contact-to-chemotherapy intervals varied by region of origin. Immigrants from the US/New Zealand/Australia or Western Europe had shorter median intervals compared to our full cohort, and immigrants from East Asia/Pacific, Latin America/Caribbean or Sub-Saharan Africa had longer median intervals such that, among symptom-detected immigrants, those from East Asia/Pacific, Latin America/Caribbean and Sub-Saharan Africa experienced 27–30-day-longer median contact-to-chemotherapy intervals than immigrants from Western Europe.

The median primary care interval was 34 days (Figure 1; Appendix B, Table A2). This interval was longer in those with stage I disease by 3–5 days and in the Champlain health region by 10–12 days. In the screen-detected group, the median primary care interval was 13–14 days shorter for those aged <50 years, and 9 days longer for those in rural remote areas. In the symptom-detected group, the median primary care interval was 5–6 days shorter for those aged <40 years or >74 years. The median surgery-to-chemotherapy interval was 58 days (Figure 1; Appendix C, Table A3). This interval was longer in those aged >74 years old by 7 days, those living very remotely rural by 8 days and those in the Champlain health region by 7 days.

**Figure 2:**
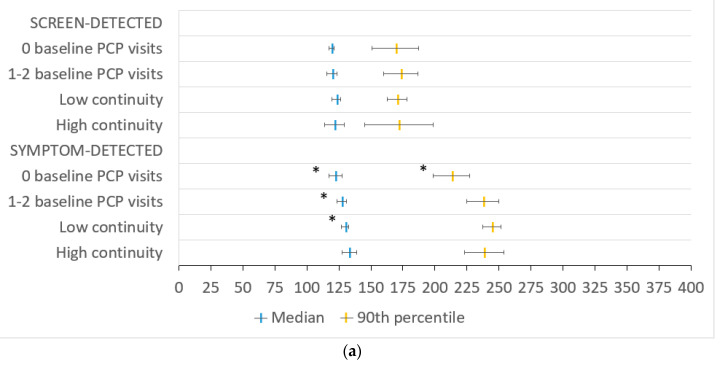

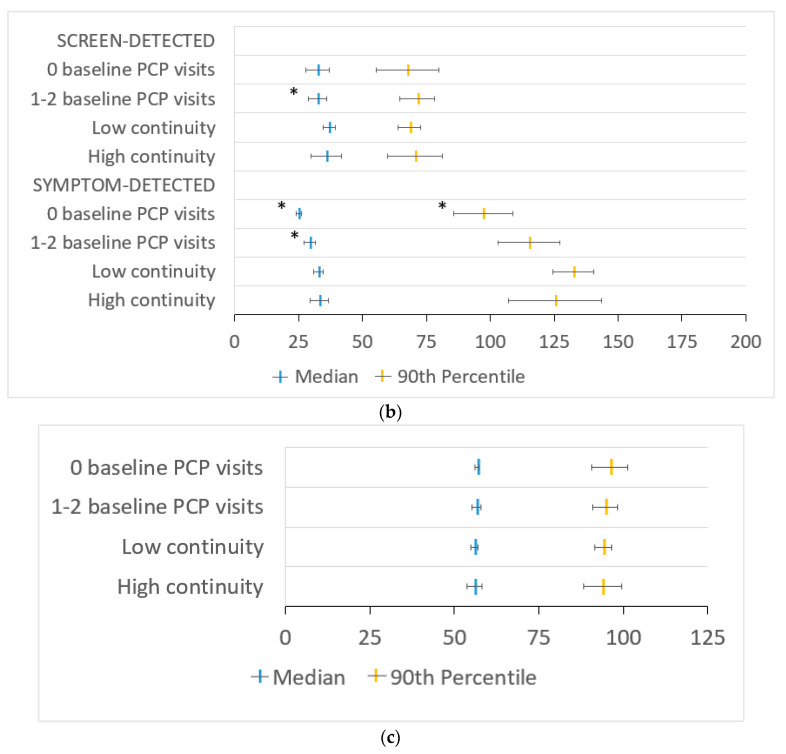
Adjusted median and 90th percentile (**a**) contact-to-chemotherapy, (**b**) primary care and (**c**) surgery-to-chemotherapy intervals in days by continuity of primary care at baseline separated by method of breast cancer detection, where applicable, with 95% confidence intervals in the entire cohort. PCP—primary care provider; low continuity—usual provider of care (UPC) index ≤ 0.75; high continuity—UPC index > 0.75. * indicates statistical significance.

In our multivariable model, symptom-detected patients with low versus high PCP continuity had a shorter median contact-to-chemotherapy interval by 3.21 days (95% CI 0.47–5.96). Symptom-detected patients with no baseline PCP visits versus high continuity had shorter median and 90th percentile intervals by 10.68 (95% CI 5.36–16.00) and 25.38 days (95% CI 11.09–39.67), respectively. Neither PCP continuity nor having few baseline PCP visits was associated with a change in the contact-to-chemotherapy interval among screen-detected patients (Figure 2a). Symptom-detected patients with no baseline PCP visits versus high PCP continuity had shorter median and 90th percentile primary care intervals by 8.04 (95% CI 5.52–10.55) and 28.14 days (95% CI 16.60–39.68), respectively. PCP continuity was not associated with a change in the primary care interval (Figure 2b). Neither PCP continuity nor having few baseline PCP visits was associated with a change in the surgery-to-chemotherapy interval (Figure 2c).

**Figure 3:**
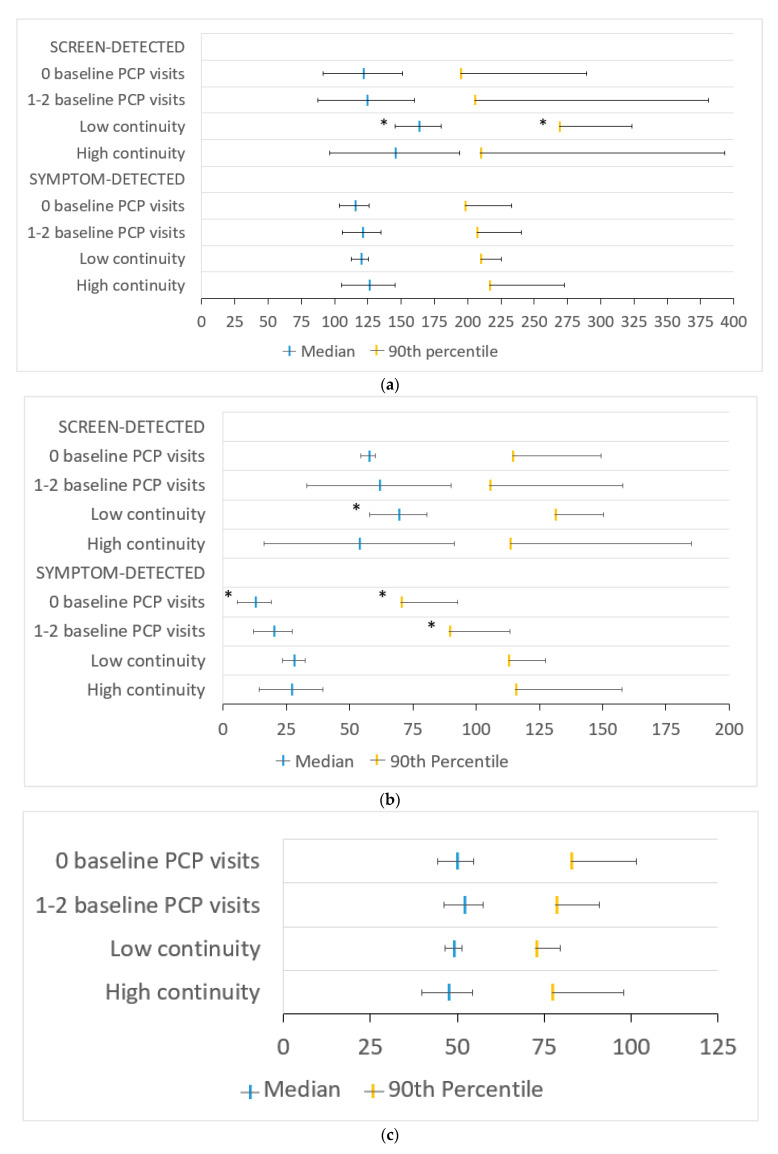
Adjusted median and 90th percentile (**a**) contact-to-chemotherapy, (**b**) primary care and (**c**) surgery-to–chemotherapy intervals in days by continuity of primary care at baseline separated by method of breast cancer detection, where applicable, with 95% confidence intervals (CIs) in the immigrant-only population. PCP—primary care provider; low continuity—usual provider of care (UPC) index ≤ 0.75; high continuity—UPC index > 0.75. * indicates statistical significance. Only the upper portion of the 95% CI is shown for 90th percentile intervals for clarity.

In screen-detected immigrants, low versus high PCP continuity was associated with longer median and 90th percentile contact-to-chemotherapy intervals by 17.43 (95% CI 0.90–34.76) and 59.37 days (95% CI 4.06–114.67), respectively (Figure 3a). The longer median interval in screen-detected immigrants was mostly accounted for by the longer median primary care sub-interval by 15.45 days (95% CI 4.00–26.90), whereas the 90th percentile primary care interval was not significantly longer in this group (17.64 days, 95% CI −1.72–37.00). In symptom-detected immigrants, having no baseline PCP visits versus high continuity was associated with shorter median and 90th percentile primary care intervals by 14.52 (95% CI 7.79–21.25) and 45.25 days (95% CI 22.49–68.01), respectively (Figure 3b). Similar to the whole cohort, there was no association between PCP continuity or having a low number of baseline PCP visits and the surgery-to-chemotherapy interval among immigrants (Figure 3c).

## 4. Discussion

In this population-based study of Ontario breast cancer patients that were diagnosed in 2007–2011, the median contact-to-chemotherapy, primary care and surgery-to-chemotherapy intervals were 126, 34 and 58 days, respectively. Other studies looked at different sub-intervals, making comparisons between studies difficult. The ICBP compared wait times for colorectal and lung cancer across several jurisdictions, including Ontario [51,52], but has not yet published findings for breast cancer wait times. Similar to other studies, we found in our unadjusted analyses that the time to breast cancer treatment was longer with increasing age [12,18], in certain Ontario health regions [23], in rural areas (for our screen-detected group) [15,18] and in those with higher comorbidity (for our symptom-detected group) [16]. Additionally, a history of mental health visits was associated with a longer time to treatment in symptom-detected patients. It may be that patients with higher age, comorbidity and/or mental health history require more preparation, counselling and/or stabilisation prior to breast cancer chemotherapy. Furthermore, in Ontario, women that were screened under 50 years of age are either considered high-risk according to the Ontario Breast Screening Program (OBSP) or are specifically referred for screening by their PCP [53], which may be associated with higher vigilance surrounding timely treatment in these younger women. Longer wait times in rural areas after positive screening may be due to the longer delays in organising follow-up tests (e.g., ultrasound) [54]. Within the immigrant population, inequities were noted based on the country of origin. Symptom-detected immigrant women from East Asia/Pacific, Latin America/Caribbean and Sub-Saharan Africa had 27–30-day-longer median contact-to-chemotherapy intervals than those from Western Europe. This is similar to another CanIMPACT study that looked at the time to diagnosis, where immigrant women in Ontario who were born in USA/New Zealand/Australia or Western Europe had the shortest adjusted time to breast cancer diagnosis and the longest adjusted time to diagnosis if they were born in East Asia/Pacific or Latin America/Caribbean [25]. Many women emigrating from East Asia/Pacific, Latin America/Caribbean and Sub-Saharan Africa would be considered racialised in Canada compared to those from Western Europe, and several international studies have found that minority race is associated with longer wait times to cancer treatment [12,13,14,19,55].

In our study, PCP continuity was not associated with the contact-to-chemotherapy interval, except in a few specific subsets of our population: in symptom-detected patients, low versus high continuity was associated with a 3-day-shorter median contact-to-chemotherapy interval, and in screen-detected immigrants, half of those with low PCP continuity waited at least 2.5 weeks longer, and 10% waited approximately 2 months longer to receive chemotherapy compared to immigrants with high PCP continuity. It is unclear why PCP continuity does not appear to play a huge role in the contact-to-chemotherapy interval length, acknowledging that a 3-day-longer median contact-to-chemotherapy interval in symptom-detected patients is not a large difference and may not be clinically meaningful. The median primary care sub-interval, i.e., the wait time from first contact with the healthcare system to the date of breast cancer consultation where primary care is thought to be mostly involved, made up only a quarter of the median contact-to-chemotherapy interval. As such, the contact-to-chemotherapy interval may be more influenced by factors outside of the realm or control of primary care. However, even the primary care interval was not associated with PCP continuity in our study. It may be that other elements of continuity of care, such as informational continuity between healthcare providers, are more important than relational continuity in determining wait times along the breast cancer care pathway. It is notable, therefore, that low relational PCP continuity was associated with such a large increase in the contact-to-chemotherapy interval among screen-detected immigrants specifically, with most of the longer interval being due to an increased primary care interval. This suggests that PCP–patient relational continuity plays an important role in influencing wait times to breast cancer consultation in immigrants with abnormal screen results. While work was done to identify wait time disparities [25,56] and barriers to cancer screening in immigrants [57,58], little has been done to explore how the handling of abnormal screening results might vary within the immigrant population. High relational PCP continuity might result in stronger patient–PCP relationships [59], allowing PCPs to provide more efficient care coordination and navigation through potentially unfamiliar healthcare institutions after abnormal screening results. Therefore, high relational PCP continuity may be particularly important for reducing wait times within this vulnerable population.

In symptom-detected patients, the contact-to-chemotherapy interval was more associated with the number of baseline PCP visits than with PCP continuity. Those with no baseline visits had shorter median and 90th percentile contact-to-chemotherapy intervals by 11 and 25 days, respectively, which was mostly due to a shorter primary care interval in this group. Patients with no baseline PCP visits may be more likely to present with later-stage disease and more alarming symptoms, which might prompt earlier referral and consultation with oncology. While this might lead to a shorter time to chemotherapy [60], this could also lead to worse outcomes [61]. This is supported by our data since those with no baseline PCP visits were more likely to be diagnosed at a later stage (stage II/III versus stage I) in our unadjusted analyses. It is also possible that PCPs are more prompted to initiate timely investigations and/or referrals for patients who they do not see often for the treatment of other conditions [35]. Those with no baseline PCP visits were more likely to live in remote rural locations or be in the lowest two income quintiles, which suggested that these groups may have a more difficult time accessing primary care.

These results lay the groundwork for future research and areas for practice and policy improvement. We showed that PCP continuity and the number of baseline PCP visits impact the contact-to-chemotherapy interval in certain populations. Future research from our team will look at the impact of these interval lengths on survival outcomes. Other future studies could examine the data record availability, completion of referral documents and/or follow-up of previously identified problems as a way to study the association between informational continuity and wait times to chemotherapy. Further work, including qualitative research with patients and providers, can explore why PCP continuity generally was not associated with the contact-to-chemotherapy interval, why immigrants with low continuity and abnormal screening experienced longer wait times to breast cancer consultation and why the longest intervals were seen for immigrants from Latin America/Caribbean, East Asia/Pacific and Sub-Saharan Africa. Other work can also look into why disparities in interval lengths were seen across the different health regions, and whether there are any specific interventions to address these disparities. It is possible that access to dedicated Breast Assessment Centres, which was shown to reduce wait times to diagnosis and sometimes treatment [21,22,62], may vary by region, and that expanding these programs to be more widely available may help to address the disparities seen across health regions. Having more structured, clear-cut referral criteria in these Breast Assessment Centres may also provide more equitable and timely care across other groups that were shown to have disparate interval lengths (e.g., age or comorbidity groups). Additionally, the median surgery-to-chemotherapy interval in our cohort was just over 8 weeks. Surgery-to-chemotherapy intervals >4 weeks were associated with higher mortality [4,5]. Therefore, shortening this interval may be an important target for breast cancer specialists and policymakers in Ontario. Policymakers should also make efforts to ensure that everyone in the population, particularly immigrants and other people who may experience challenges navigating the system, has a regular PCP.

Our results should be interpreted in light of certain limitations. Most notably, we were unable to examine the patient interval (from first symptoms to first healthcare presentation). PCP continuity may have a large part to play in decreasing the patient interval. Unfortunately, it is not possible to capture this interval using health administrative data. Second, since we used health administrative data in our study, the information on some variables, such as primary language, race and marital status, were not available. Third, we did not capture ED involvement in our study. While the number of breast cancer patients first presenting to the ED is small (~3–4%) [34,63], ED presentation may mediate the relationship between baseline PCP continuity/number of PCP visits and the contact-to-chemotherapy interval. Fourth, we did not explore the route to screening (through the OBSP versus through the PCP) or whether this impacted the contact-to-chemotherapy interval. Fifth, the CanIMPACT cohort that was used in this study included patients that were diagnosed in 2007–2011. While breast cancer treatment principles have not changed greatly since 2011 [64], and there has not been any major primary care reform in Ontario since that time [65], we need to consider that the effect of PCP continuity on the contact-to-chemotherapy interval may have changed from when these patients were treated.

## 5. Conclusions

We found that baseline PCP continuity was not associated with the contact-to-chemotherapy interval in the Ontario breast cancer population except in specific groups: we found that high baseline PCP continuity was associated with shorter wait times to breast cancer consultation and receiving adjuvant chemotherapy in screen-detected immigrants, and marginally increased wait times to chemotherapy in symptom-detected patients. Additionally, having no baseline PCP visits was associated with increased wait times to breast cancer consultation and receiving adjuvant chemotherapy. This highlights the importance of having access to PCPs and ensuring that immigrants and others who may have difficulty navigating the healthcare system have high PCP continuity.

## Figures and Tables

**Table 1:** Baseline characteristics according to continuity of care at baseline.

	Total *n* = 12,781	PCP Continuity				*p*-Value
		0 Visits	1–2 Visits	UPC ≤ 0.75 (Low)	UPC > 0.75 (High)	
Total		800 (100%)	1536 (100%)	3914 (100%)	6531 (100%)	
Age (Categorical)						
<40 years	1102 (8.6%)	69 (8.6%)	142 (9.2%)	457 (11.7%)	434 (6.6%)	<0.001
40–49 years	3481 (27.2%)	226 (28.3%)	499 (32.5%)	1237 (31.6%)	1519 (23.3%)	
50–59 years	4225 (33.1%)	302 (37.8%)	533 (34.7%)	1251 (32.0%)	2139 (32.8%)	
60–69 years	3045 (23.8%)	176 (22.0%)	309 (20.1%)	779 (19.9%)	1781 (27.3%)	
70–74 years	607 (4.7%)	15 (1.9%)	37 (2.4%)	126 (3.2%)	429 (6.6%)	
>74 years	321 (2.5%)	12 (1.5%)	16 (1.0%)	64 (1.6%)	229 (3.5%)	
Urban/Rural Residence						
Urban	11,189 (87.5%)	664 (83.0%)	1283 (83.5%)	3549 (90.7%)	5693 (87.2%)	<0.001
Rural	699 (5.5%)	45 (5.6%)	108 (7.0%)	149 (3.8%)	397 (6.1%)	
Rural—remote	596 (4.7%)	62 (7.8%)	94 (6.1%)	119 (3.0%)	321 (4.9%)	
Rural—very remote	292–297 (2.3%)	25–30 (3.1–3.8%)	50–55 (3.3–3.6%)	93–98 (2.4–2.5%)	115–120 (1.8%)	
Rural—unknown	*	*	*	*	*	
Unknown	*	*	*	*	*	
Immigration Status						
Long-term residents	11,075 (86.7%)	681 (85.1%)	1373 (89.4%)	3281 (83.8%)	5740 (87.9%)	<0.001
Immigrants	1706 (13.3%)	119 (14.9%)	163 (10.6%)	633 (16.2%)	791 (12.1%)	
Immigrant Region of Origin						
East Asia and Pacific	544 (4.3%)	34 (4.3%)	51 (3.3%)	191 (4.9%)	268 (4.1%)	<0.001
Eastern Europe and Central Asia	286 (2.2%)	29 (3.6%)	43 (2.8%)	96 (2.5%)	118 (1.8%)	
Latin America and Caribbean	239 (1.9%)	13 (1.6%)	16 (1.0%)	94 (2.4%)	116 (1.8%)	
Middle East and North Africa	145 (1.1%)	16 (2.0%)	6 (0.4%)	55 (1.4%)	68 (1.0%)	
South Asia	270 (2.1%)	12 (1.5%)	16 (1.0%)	111 (2.8%)	131 (2.0%)	
Sub-Saharan Africa	87 (0.7%)	3–7 (0.4–0.9%)	6–10 (0.4–0.7%)	44 (1.1%)	30 (0.5%)	
USA/New Zealand/Australia	37 (0.3%)	*	5–9 (0.3–0.6%)	14 (0.4%)	12 (0.2%)	
Western Europe	98 (0.8%)	6 (0.8%)	16 (1.0%)	28 (0.7%)	48 (0.7%)	
Neighbourhood Income Quintile						
1 (lowest)	2020 (15.8%)	150 (18.8%)	227 (14.8%)	597 (15.3%)	1046 (16.0%)	<0.001
2	2384 (18.7%)	191 (23.9%)	276 (18.0%)	696 (17.8%)	1221 (18.7%)	
3	2523 (19.7%)	140–144 (17.5–18.0%)	274–278 (17.8–18.1%)	807 (20.6%)	1298 (19.9%)	
4	2819 (22.1%)	153 (19.1%)	351 (22.9%)	873 (22.3%)	1442 (22.1%)	
5 (highest)	2994 (23.4%)	160 (20.0%)	401 (26.1%)	928 (23.7%)	1505 (23.0%)	
Unknown	41 (0.3%)	*	*	13 (0.3%)	19 (0.3%)	
Comorbidity Burden						
0–5 ADGs	7287 (57.0%)	788 (98.5%)	1472 (95.8%)	1773 (45.3%)	3254 (49.8%)	<0.001
6–9 ADGs	4425 (34.6%)	10–14 (1.3–1.8%)	55–59 (3.6–3.8%)	1661 (42.4%)	2695 (41.3%)	
10+ ADGs	1069 (8.4%)	*	*	480 (12.3%)	582 (8.9%)	
History of Mental Health Visits						
Yes	4127 (32.3%)	18 (2.3%)	149 (9.7%)	1486 (38.0%)	2474 (37.9%)	<0.001
Cancer Detection Method						
Screening	2916 (22.8%)	164 (20.5%)	328 (21.4%)	776 (19.8%)	1648 (25.2%)	<0.001
Symptomatic	9865 (77.2%)	636 (79.5%)	1208 (78.6%)	3138 (80.2%)	4883 (74.8%)	
Stage						
Stage I	2839 (22.2%)	140 (17.5%)	328 (21.4%)	886 (22.6%)	1485 (22.7%)	0.017
Stage II	7311 (57.2%)	470 (58.8%)	889 (57.9%)	2251 (57.5%)	3701 (56.7%)	
Stage III	2631 (20.6%)	190 (23.8%)	319 (20.8%)	777 (19.9%)	1345 (20.6%)	
Primary Care Practice Model						
Straight FFS	1887 (14.8%)	301 (37.6%)	277 (18.0%)	542 (13.8%)	767 (11.7%)	<0.001
Enhanced FFS	6281 (49.1%)	228 (28.5%)	553 (36.0%)	2036 (52.0%)	3464 (53.0%)	
Capitation	2235 (17.5%)	110 (13.8%)	303 (19.7%)	654 (16.7%)	1168 (17.9%)	
Team-based capitation	2206 (17.3%)	123 (15.4%)	369 (24.0%)	642 (16.4%)	1072 (16.4%)	
Other	172 (1.3%)	38 (4.8%)	34 (2.2%)	40 (1.0%)	60 (0.9%)	
Health Region						
1 Erie St. Clair	713 (5.6%)	47 (5.9%)	88 (5.7%)	221 (5.6%)	357 (5.5%)	<0.001
2 South West	992 (7.8%)	55 (6.9%)	145 (9.4%)	242 (6.2%)	550 (8.4%)	
3 Waterloo Wellington	654 (5.1%)	59 (7.4%)	125 (8.1%)	140 (3.6%)	330 (5.1%)	
4 Hamilton Niagara Haldimand Brant	1468 (11.5%)	101 (12.6%)	198 (12.9%)	413 (10.6%)	756 (11.6%)	
5 Central West	543 (4.2%)	25 (3.1%)	30 (2.0%)	197 (5.0%)	291 (4.5%)	
6 Mississauga Halton	750 (5.9%)	47 (5.9%)	67 (4.4%)	280 (7.2%)	356 (5.5%)	
7 Toronto Central	1061 (8.3%)	65 (8.1%)	121 (7.9%)	357 (9.1%)	518 (7.9%)	
8 Central	1784 (14.0%)	72 (9.0%)	152 (9.9%)	626 (16.0%)	934 (14.3%)	
9 Central East	1710 (13.4%)	90 (11.3%)	177 (11.5%)	495 (12.6%)	948 (14.5%)	
10 South East	520 (4.1%)	49 (6.1%)	81 (5.3%)	125 (3.2%)	265 (4.1%)	
11 Champlain	1335 (10.4%)	108 (13.5%)	183 (11.9%)	444 (11.3%)	600 (9.2%)	
12 North Simcoe Muskoka	518–522 (4.1%)	12–16 (1.5–2.0%)	70–74 (4.6–4.8%)	165–169 (4.2–4.3%)	266–270 (4.1%)	
13 North East	478 (3.7%)	44 (5.5%)	64 (4.2%)	129 (3.3%)	241 (3.7%)	
14 North West	252 (2.0%)	24 (3.0%)	34 (2.2%)	78 (2.0%)	116 (1.8%)	
Unknown	*	*	*	*	*	

* denotes too few cases to report. Ranges are provided in associated rows or columns to prevent the reidentification of small cells as per the ICES policy. Note: PCP—primary care provider, UPC—usual provider of care index, ADG—Aggregated Diagnosis Groups, FFS—fee for service.

**Table 2:** Baseline characteristics according to median contact-to-adjuvant-chemotherapy interval (in days) stratified by screened versus symptomatic detection.

	Total *n* = 12,781	Contact-to-Adjuvant-Chemotherapy Interval in Days					
		Screened *n* = 2916 (22.8%)			Symptomatic *n* = 9865 (77.2%)		
		Median (IQR)	90th Percentile	*p*-Value *	Median (IQR)	90th Percentile	*p*-Value *
Total		125 (103, 154)	185		127 (99, 171)	228	
Age (Categorical)				<0.0001			<0.0001
<40 years	1102 (8.6%)	107 (85, 124)	189		115 (90, 155)	205	
40–49 years	3481 (27.2%)	115 (93, 147)	178		126 (99, 170)	228	
50–59 years	4225 (33.1%)	124 (103, 154)	187		128 (101, 175)	233	
60–69 years	3045 (23.8%)	126 (105, 155)	184		132 (103, 176)	231	
70–74 years	607 (4.7%)	125 (104, 158)	185		138 (108, 179)	224	
>74 years	321 (2.5%)	137 (118, 162)	187		134 (104, 175)	221	
Urban/Rural Residence				<0.0001			0.4999
Urban	11,189 (87.5%)	123 (102, 153)	182		127 (99, 170)	227	
Rural	699 (5.5%)	127 (110, 159)	189		125 (102, 175)	223	
Rural—remote	596 (4.7%)	134 (110, 164)	194		127 (98, 173)	225	
Rural—very remote	292–297 (2.3%)	153 (122, 184)	231		132 (104, 182)	259	
Rural—unknown	≤5	**	**		**	**	
Unknown	≤5	**	**		**	**	
Immigration Status				0.1425			0.0008
Long-term residents	11,075 (86.7%)	125 (103, 154)	184		126 (99, 170)	227	
Immigrants	1706 (13.3%)	129 (104, 161)	194		133 (104, 175)	231	
Immigrant Region of Origin				0.9288			0.0085
East Asia and Pacific	544 (4.3%)	135 (106, 161)	191		138 (104, 175)	231	
Eastern Europe and Central Asia	286 (2.2%)	135 (102, 167)	191		127 (100, 173)	230	
Latin America and Caribbean	239 (1.9%)	129 (116, 154)	258		141 (108, 179)	241	
Middle East and North Africa	145 (1.1%)	124 (104, 147)	191		134 (108, 181)	218	
South Asia	270 (2.1%)	126 (98, 160)	194		134 (109, 169)	217	
Sub-Saharan Africa	87 (0.7%)	137 (103, 155)	163		139 (106, 180)	225	
USA/New Zealand/Australia	37 (0.3%)	119 (103, 148)	162		119 (100, 178)	231	
Western Europe	98 (0.8%)	123 (105, 176)	203		111 (94, 144)	231	
Neighbourhood Income Quintile				0.1196			0.1620
1 (lowest)	2020 (15.8%)	128 (106, 160)	188		130 (100, 175)	226	
2	2384 (18.7%)	125 (104, 155)	181		128 (100, 170)	231	
3	2523 (19.7%)	125 (104, 155)	183		127 (101, 174)	225	
4	2819 (22.1%)	127 (103, 153)	186		126 (99, 168)	226	
5 (highest)	2994 (23.4%)	122 (100, 151)	184		125 (98, 170)	231	
Unknown	41 (0.3%)	170 (119, 226)	247		143 (102, 182)	234	
Comorbidity Burden				0.7763			<0.0001
0–5 ADGs	7287 (57.0%)	124 (104, 153)	183		123 (98, 166)	219	
6–9 ADGs	4425 (34.6%)	126 (103, 155)	189		133 (103, 178)	238	
10+ ADGs	1069 (8.4%)	126 (104, 158)	182		135 (104, 183)	245	
History of Mental Health Visits				0.9609			<0.0001
Yes	4127 (32.3%)	124 (102, 155)	191		132 (103, 176)	233	
No	8654 (67.7%)	126 (104, 154)	183		125 (98, 169)	225	
Stage				0.0010			<0.0001
Stage I	2839 (22.2%)	128 (105, 158)	188		136 (105, 185)	242	
Stage II	7311 (57.2%)	125 (103, 154)	184		127 (100, 169)	225	
Stage III	2631 (20.6%)	119 (100, 146)	182		119 (93, 162)	219	
Primary Care Model				0.0373			0.0012
Straight FFS	1887 (14.8%)	127 (104, 152)	182		126 (100, 169)	221	
Enhanced FFS	6281 (49.1%)	127 (104, 159)	190		128 (100, 172)	230	
Capitation	2235 (17.5%)	121 (102, 153)	180		127 (100, 175)	233	
Team-based capitation	2206 (17.3%)	121 (101, 149)	182		122 (97, 166)	228	
Other	172 (1.3%)	126 (108, 157)	190		117 (91, 155)	203	
Primary Care Enrolment Status				0.7247			0.6580
Rostered	10,900 (85.3%)	125 (103, 155)	185		127 (99, 171)	230	
Not rostered	1881 (14.7%)	127 (104, 152)	183		127 (100, 169)	221	
Health Region				<0.0001			<0.0001
1 Erie St. Clair	713 (5.6%)	118 (99, 142)	179		120 (92, 157)	208	
2 South West	992 (7.8%)	138 (113, 167)	200		133 (103, 172)	227	
3 Waterloo Wellington	654 (5.1%)	119 (98, 141)	167		112 (91, 150)	207	
4 Hamilton Niagara Haldimand Brant	1468 (11.5%)	118 (100, 140)	170		116 (96, 155)	213	
5 Central West	543 (4.2%)	120 (99, 150)	182		126 (99, 171)	223	
6 Mississauga Halton	750 (5.9%)	120 (96, 154)	196		124 (96, 173)	234	
7 Toronto Central	1061 (8.3%)	126 (106, 155)	184		134 (105, 185)	247	
8 Central	1784 (14.0%)	124 (101, 154)	188		128 (101, 174)	231	
9 Central East	1710 (13.4%)	114 (95, 146)	179		127 (98, 171)	220	
10 South East	520 (4.1%)	126 (106, 159)	183		120 (99, 157)	217	
11 Champlain	1335 (10.4%)	144 (121, 169)	189		148 (120, 189)	249	
12 North Simcoe Muskoka	518–522 (4.1%)	126 (103, 162)	176		122 (102, 176)	237	
13 North East	478 (3.7%)	118 (98, 147)	190		117 (88, 160)	216	
14 North West	252 (2.0%)	143 (108, 161)	198		128 (92, 173)	231	
Unknown	≤5	**	**		**	**	

* *p*-values calculated for median values. ** values suppressed due to small cells. Note: ADG—Aggregated Diagnosis Groups, FFS—fee for service.

## Data Availability

The dataset from this study is held securely in a coded form at ICES. While data-sharing agreements prohibit ICES from making the dataset publicly available, access may be granted to those who meet pre-specified criteria for confidential access, available at www.ices.on.ca/DAS (accessed on 19 December 2019). The full dataset creation plan is available from the authors upon request.

## Appendix A

**Table A1:** Data sources that were used to obtain the data elements for variable creation.

Data Source	Data Elements
Ontario Cancer Registry (OCR)	Date of breast cancer diagnosis, age at diagnosis, sex, other cancer diagnoses, cancer stage
Registered Persons Database (RPDB)	Postal code at time of diagnosis, health region
2006 Statistics Canada Census and Postal Code Conversion File Plus, version 5C	Rurality, neighborhood income quintile
Immigration Refugee and Citizenship Canada permanent resident (IRCC-PR) database	Immigration status
Ontario Health Insurance Plan (OHIP)	Number of PCP visits (billed encounters) total and per provider, reasons for visits, diagnostic codes, chemotherapy receipt, start of adjuvant chemotherapy
ICES Physician Database	Physician specialty
Client Agency Program Enrolment database (CAPE) and Corporate Provider Database	Primary care enrolment model
Canadian Institute for Health Information: Discharge Abstract Database (DAD) and Same-Day Surgery (SDS) database	Diagnosis codes, surgery receipt
Cancer Activity Level Reporting (ALR) database	Date of radiotherapy receipt

## Appendix B

**Table A2:** Baseline characteristics according to the median and 90th percentile primary care intervals in days stratified by the method of detection.

	Total *n* = 12,781	Primary Care Interval in Days (from First Contact to First Oncology Visit)					
		Screened *n* = 2916 (22.8%)			Symptomatic *n* = 9865 (77.2%)		
		Median (IQR)	90th Percentile	Kruskal–Wallis *p*-value *	Median (IQR)	90th Percentile	Kruskal–Wallis *p*-value *
Total		34 (21, 50)	72		34 (17, 62)	111	
Age (Categorical)				<0.0001			<0.0001
<40 years	1102 (8.6%)	20 (18, 34)	51		29 (14, 56)	101	
40–49 years	3481 (27.2%)	21 (9, 42)	85		35 (19, 64)	117	
50–59 years	4225 (33.1%)	34 (21, 51)	74		35 (16, 64)	113	
60–69 years	3045 (23.8%)	35 (22, 50)	70		34 (16, 63)	108	
70–74 years	607 (4.7%)	35 (20, 50)	67		34 (17, 59)	99	
>74 years	321 (2.5%)	37 (21, 60)	79		28 (14, 55)	83	
Urban/Rural Residence				<0.0001			0.0078
Urban	11,189 (87.5%)	34 (20, 49)	70		34 (17, 63)	110	
Rural	699 (5.5%)	36 (21, 50)	74		35 (17, 62)	113	
Rural—remote	596 (4.7%)	41 (25, 60)	84		32 (15, 56)	106	
Rural—very remote	292–297 (2.3%)	43 (26, 70)	91		28 (12, 58)	113	
Rural—unknown	≤5	**	**		**	**	
Unknown	≤5	**	**		**	**	
Immigration Status				0.4322			0.9899
Long-term residents	11,075 (86.7%)	34 (21, 50)	71		34 (17, 62)	111	
Immigrants	1706 (13.3%)	33 (17, 54)	76		34 (17, 63)	109	
							
Immigrant Region of Origin				0.2853			0.0783
East Asia and Pacific	544 (4.3%)	33 (17, 62)	94		33 (15, 63)	112	
Eastern Europe and Central Asia	286 (2.2%)	27 (9, 51)	56		33 (16, 62)	112	
Latin America and Caribbean	239 (1.9%)	36 (21, 53)	84		41 (19, 72)	107	
Middle East and North Africa	145 (1.1%)	41 (16, 51)	62		42 (19, 75)	121	
South Asia	270 (2.1%)	30 (15, 44)	78		28 (15, 53)	95	
Sub-Saharan Africa	87 (0.7%)	41 (23, 55)	69		37 (18, 69)	90	
USA/New Zealand/Australia	37 (0.3%)	36 (28, 76)	105		36 (15, 60)	117	
Western Europe	98 (0.8%)	38 (24, 67)	113		29 (14, 49)	87	
Neighbourhood Income Quintile				0.7635			0.7172
1 (lowest)	2020 (15.8%)	35 (21, 54)	75		34 (17, 62)	105	
2	2384 (18.7%)	34 (21, 52)	71		34 (17, 63)	106	
3	2523 (19.7%)	35 (21, 51)	72		34 (17, 63)	108	
4	2819 (22.1%)	35 (21, 49)	70		34 (16, 59)	107	
5 (highest)	2994 (23.4%)	34 (21, 49)	70		34 (17, 64)	122	
Unknown	41 (0.3%)	42 (25, 89)	102		42 (25, 63)	112	
Comorbidity Burden				0.9419			<0.0001
0–5 ADGs	7287 (57.0%)	35 (21, 50)	71		33 (16, 59)	105	
6–9 ADGs	4425 (34.6%)	34 (21, 51)	73		36 (18, 66)	115	
10+ ADGs	1069 (8.4%)	35 (21, 53)	71		34 (17, 63)	120	
History of Mental Health Visits				0.6662			0.0007
Yes	4127 (32.3%)	34 (21, 51)	72		35 (18, 65)	115	
No	8654 (67.7%)	35 (21, 50)	72		33 (16, 61)	108	
Stage				<0.0001			<0.0001
Stage I	2839 (22.2%)	37 (23, 54)	76		39 (21, 71)	122	
Stage II	7311 (57.2%)	33 (20, 49)	71		34 (17, 61)	106	
Stage III	2631 (20.6%)	32 (19, 48)	69		29 (14, 56)	107	
Primary Care Practice Model				0.5489			0.0078
Straight FFS	1887 (14.8%)	36 (20, 52)	71		32 (15, 62)	103	
Enhanced FFS	6281 (49.1%)	35 (21, 51)	71		35 (17, 62)	108	
Capitation	2235 (17.5%)	35 (21, 50)	73		35 (17, 68)	119	
Team-based capitation	2206 (17.3%)	33 (20, 50)	72		33 (17, 61)	115	
Other	172 (1.3%)	35 (25, 48)	63		27 (12, 49)	77	
Primary Care Enrolment Status				0.4377			0.0256
Rostered	10,900 (85.3%)	34 (21, 50)	72		34 (17, 62)	112	
Not rostered	1881 (14.7%)	36 (20, 52)	71		32 (15, 62)	103	
Health Region				<0.0001			<0.0001
1 Erie St. Clair	713 (5.6%)	35 (21, 49)	71		40 (21, 68)	116	
2 South West	992 (7.8%)	44 (27, 67)	89		40 (20, 69)	119	
3 Waterloo Wellington	654 (5.1%)	33 (20, 44)	57		27 (14, 51)	105	
4 Hamilton Niagara Haldimand Brant	1468 (11.5%)	29 (15, 43)	57		32 (16, 55)	99	
5 Central West	543 (4.2%)	39 (25, 50)	63		33 (17, 52)	90	
6 Mississauga Halton	750 (5.9%)	31 (17, 49)	78		35 (16, 65)	116	
7 Toronto Central	1061 (8.3%)	34 (19, 55)	76		34 (16, 67)	120	
8 Central	1784 (14.0%)	29 (17, 47)	69		31 (15, 61)	114	
9 Central East	1710 (13.4%)	29 (18, 41)	52		32 (15, 58)	104	
10 South East	520 (4.1%)	40 (25, 61)	80		31 (17, 56)	103	
11 Champlain	1335 (10.4%)	44 (30, 57)	70		46 (28, 74)	127	
12 North Simcoe Muskoka	518–522 (4.1%)	27 (17, 43)	68		27 (15, 58)	110	
13 North East	478 (3.7%)	32 (21, 43)	63		27 (12, 53)	91	
14 North West	252 (2.0%)	56 (37, 77)	95		35 (14, 63)	107	
Unknown	≤5	**	**		**	**	

* *p*-values calculated for median values. ** values suppressed due to small cells. Note: ADG—Aggregated Diagnosis Groups, FFS—fee for service.

## Appendix C

**Table A3:** Baseline characteristics according to median surgery-to-chemotherapy interval in days.

	Total *n* = 12,781	Surgery-to-Adjuvant-Chemotherapy Interval in Days		
		Median (IQR)	90th percentile	Kruskal–Wallis *p*-value *
Total		58 (46, 74)	93	
Age (Categorical)				<0.0001
<40 years	1102 (8.6%)	52 (41, 68)	87	
40–49 years	3481 (27.2%)	56 (44, 71)	91	
50–59 years	4225 (33.1%)	58 (46, 74)	93	
60–69 years	3045 (23.8%)	60 (48, 76)	96	
70–74 years	607 (4.7%)	62 (49, 81)	98	
>74 years	321 (2.5%)	65 (49, 81)	105	
Urban/Rural Residence				<0.0001
Urban	11,189 (87.5%)	57 (45, 73)	92	
Rural	699 (5.5%)	62 (48, 77)	94	
Rural—remote	596 (4.7%)	63 (50, 79)	98	
Rural—very remote	292–297 (2.3%)	66 (49, 86)	110	
Rural—unknown	≤5	**	**	
Unknown	≤5	**	**	
Immigration Status				0.2876
Long-term residents	11,075 (86.7%)	58 (46, 74)	93	
Immigrants	1706 (13.3%)	57 (44, 75)	96	
Immigrant Region of Origin				0.1119
East Asia and Pacific	544 (4.3%)	57 (43, 77)	98	
Eastern Europe and Central Asia	286 (2.2%)	56 (45, 70)	88	
Latin America and Caribbean	239 (1.9%)	59 (46, 77)	107	
Middle East and North Africa	145 (1.1%)	59 (43, 76)	91	
South Asia	270 (2.1%)	60 (46, 78)	102	
Sub-Saharan Africa	87 (0.7%)	53 (43, 70)	99	
USA/New Zealand/Australia	37 (0.3%)	52 (38, 72)	91	
Western Europe	98 (0.8%)	55 (42, 67)	87	
Neighbourhood Income Quintile				0.0456
1 (lowest)	2020 (15.8%)	57 (45, 75)	93	
2	2384 (18.7%)	58 (46, 75)	94	
3	2523 (19.7%)	58 (46, 76)	96	
4	2819 (22.1%)	58 (45, 73)	92	
5 (highest)	2994 (23.4%)	57 (45, 72)	91	
Unknown	41 (0.3%)	62 (48, 85)	104	
Comorbidity Burden				0.0561
0–5 ADGs	7287 (57.0%)	57 (46, 73)	92	
6–9 ADGs	4425 (34.6%)	58 (46, 74)	94	
10+ ADGs	1069 (8.4%)	59 (45, 78)	99	
History of Mental Health Visits				0.0595
Yes	4127 (32.3%)	58 (46, 75)	94	
No	8654 (67.7%)	57 (45, 74)	92	
Stage				<0.0001
Stage I	2839 (22.2%)	60 (48, 76)	98	
Stage II	7311 (57.2%)	58 (47, 75)	93	
Stage III	2631 (20.6%)	53 (41, 69)	87	
Primary Care Practice Model				0.1546
Straight FFS	1887 (14.8%)	57 (45, 74)	96	
Enhanced FFS	6281 (49.1%)	58 (45, 74)	94	
Capitation	2235 (17.5%)	57 (45, 73)	92	
Team-based capitation	2206 (17.3%)	59 (47, 73)	91	
Other	172 (1.3%)	56 (42, 75)	97	
Primary Care Enrolment Status				0.5820
Rostered	10,900 (85.3%)	58 (46, 74)	93	
Not rostered	1881 (14.7%)	57 (45, 74)	96	
Health Region				<0.0001
1 Erie St. Clair	713 (5.6%)	50 (40, 69)	86	
2 South West	992 (7.8%)	63 (52, 79)	98	
3 Waterloo Wellington	654 (5.1%)	52 (41, 68)	83	
4 Hamilton Niagara Haldimand Brant	1468 (11.5%)	56 (45, 70)	86	
5 Central West	543 (4.2%)	58 (44, 74)	98	
6 Mississauga Halton	750 (5.9%)	55 (43, 71)	92	
7 Toronto Central	1061 (8.3%)	57 (45, 71)	96	
8 Central	1784 (14.0%)	56 (43, 73)	93	
9 Central East	1710 (13.4%)	57 (45, 74)	94	
10 South East	520 (4.1%)	59 (48, 73)	91	
11 Champlain	1335 (10.4%)	65 (53, 79)	96	
12 North Simcoe Muskoka	518–522 (4.1%)	63 (51, 78)	93	
13 North East	478 (3.7%)	61 (45, 77)	107	
14 North West	252 (2.0%)	52 (36, 72)	97	
Unknown	≤5	**	**	

* *p*-values calculated for median values. ** values suppressed due to small cells. Note: ADG—Aggregated Diagnosis Groups, FFS—fee for service.
